# Native opportunities to stop hypertension: study protocol for a randomized controlled trial among urban American Indian and Alaska Native adults with hypertension

**DOI:** 10.3389/fpubh.2023.1117824

**Published:** 2023-06-02

**Authors:** Ka’imi Sinclair, Cassandra J. Nguyen, Marianna S. Wetherill, Katie Nelson, Alexandra M. Jackson, Tori Taniguchi, Valarie Blue Bird Jernigan, Dedra Buchwald

**Affiliations:** ^1^Institute for Research and Education to Advance Community Health, Washington State University, Seattle, WA, United States; ^2^Nutrition Department at University of California, Davis, Davis, CA, United States; ^3^Department of Health Promotion Sciences, Hudson College of Public Health, University of Oklahoma Health Sciences Center, Tulsa, OK, United States; ^4^Public Health Department, Pacific University, Forest Grove, OR, United States; ^5^Center for Indigenous Health Research and Policy, Center for Health Sciences, Oklahoma State University, Tulsa, OK, United States

**Keywords:** American Indian/Alaska Native, dietary approach to stop hypertension, cardiovascular disease, blood pressure, health disparities, urban health, telenutrition, healthy diet

## Abstract

**Introduction:**

American Indian and Alaska Native (AI/AN) adults experience disproportionate cardiovascular disease (CVD) morbidity and mortality compared to other races, which may be partly attributable to higher burden of hypertension (HTN). Dietary Approaches to Stop Hypertension (DASH) is a high-impact therapeutic dietary intervention for primary and secondary prevention of CVD that can contribute to significant decreases in systolic blood pressure (BP). However, DASH-based interventions have not been tested with AI/AN adults, and unique social determinants of health warrant independent trials. This study will assess the effectiveness of a DASH-based intervention, called Native Opportunities to Stop Hypertension (NOSH), on systolic BP among AI/AN adults in three urban clinics.

**Methods:**

NOSH is a randomized controlled trial to test the effectiveness of an adapted DASH intervention compared to a control condition. Participants will be aged ≥18 years old, self-identify as AI/AN, have physician-diagnosed HTN, and have elevated systolic BP (≥ 130 mmHg). The intervention includes eight weekly, tailored telenutrition counseling sessions with a registered dietitian on DASH eating goals. Intervention participants will be provided $30 weekly and will be encouraged to purchase DASH-aligned foods. Participants in the control group will receive printed educational materials with general information about a low-sodium diet and eight weekly $30 grocery orders. All participants will complete assessments at baseline, after the 8-week intervention, and again 12 weeks post-baseline. A sub-sample of intervention participants will complete an extended support pilot study with assessments at 6- and 9-months post-baseline. The primary outcome is systolic BP. Secondary outcomes include modifiable CVD risk factors, heart disease and stroke risk scores, and dietary intake.

**Discussion:**

NOSH is among the first randomized controlled trials to test the impact of a diet-based intervention on HTN among urban AI/AN adults. If effective, NOSH has the potential to inform clinical strategies to reduce BP among AI/AN adults.

**Clinical trials registration:**

https://clinicaltrials.gov/ct2/show/NCT02796313, Identifier NCT02796313.

## 1. Introduction

American Indian and Alaska Native (AI/AN) people experience a disproportionate prevalence of cardiovascular disease (CVD) morbidity and mortality compared to other races (1, 2). CVD is the term for all types of diseases that affect the heart or blood vessels, including coronary heart disease (CHD), which can cause heart attack, stroke, heart failure, and peripheral artery disease (3). CHD prevalence rates among AI/AN people are greater than 12% (4) and may be underreported by 21% (5). The prevalence of CHD is particularly high among younger AI/AN people where more than one-third of deaths occur before the age of 65 years (6). Similarly, deaths due to stroke are the highest among younger AI/AN adults <45 years of age compared with other racial and ethnic groups in the United States (4).

Hypertension (HTN) is a major independent risk factor for the development of CHD and stroke (7, 8). A healthy diet can improve HTN management with or without medication (9–11) while also conferring other benefits, such as weight loss and healthier lipid profiles (12–15). Dietary Approaches to Stop Hypertension (DASH) is a high-impact therapeutic dietary intervention for primary and secondary prevention of CVD and is recognized as an effective dietary intervention to reduce blood pressure (BP) (16, 17). The DASH diet encourages low intakes of sodium and saturated fat paired with high intakes of fruits and vegetables. In a randomized trial, the DASH diet lowered systolic BP among participants with both borderline and clinical HTN (18). Despite a high prevalence of CVD-related morbidity and mortality and the effectiveness of the DASH diet to improve BP control across multiple studies, AI/AN people are noticeably absent in the research conducted to date.

AI/AN people interact with a unique healthcare system that may, in part, contribute to CVD inequities. The United States government provides healthcare to members of federally recognized Tribes through a treaty-based responsibility that has been filled by the Indian Health Service (IHS) since 1955. However, in reality, AI/AN people obtain healthcare through a fragmented process that includes IHS, Tribal, and urban Indian healthcare facilities, as well as public, private, and state-operated health initiatives. Notably, all IHS facilities are located on or near reservations, making many of them geographically inaccessible to most AI/AN people living in urban areas (19). Unsurprisingly, AI/AN adults often report more difficulties in healthcare access than White adults and have lower rates of healthcare utilization (20). Further, the IHS has been chronically underfunded, with $3,332 spent per patient per year in comparison to $9,207 per patient for federal health care nationwide (21). Despite the efforts of many Native nations to exert sovereignty and provide culturally-relevant care that incorporates both Western and traditional medicines, the complex configuration of healthcare systems, along with the policy and regulatory environments in which they operate, can adversely affect the ability of AI/AN people to obtain high-quality healthcare in Tribal and urban settings (22, 23).

Nationwide, AI/AN communities have lost access to traditional foods (24), which were historically nutrient dense and minimally processed. Furthermore, as AI/AN adults often have lower incomes than the general population, their ability to purchase healthy foods is limited (25, 26). AI/AN households are at greater risk of experiencing food insecurity, wherein a nutritionally-balanced diet is not geographically available or financially accessible, with prevalence rates ranging from 16 to 80% (27). Food insecurity is related to greater risk of HTN (27–30), and dependence on processed foods that increase sodium intake can further elevate risk (31–34). These barriers to access of healthful and affordable food options can, in part, be addressed through education and the increasingly common clinic-based food assistance programs that can involve medically-tailored groceries or food vouchers (35, 36).

Three key attributes are important for an intervention to successfully address HTN disparities experienced by AI/AN adults. First, an intervention must consider the needs of AI/AN adults in urban settings. Few studies have examined rates of HTN in urban AI/AN adults (37). Notably, most observational and intervention studies of CVD have focused on reservation-based AI/AN people, who receive care through the IHS and Tribally-run clinics, even though 71% of AI/AN adults live in urban areas (38). This urban population is an invisible minority (39), with high rates of disease and disability, low rates of healthcare usage (26, 40–42), and elevated risk of food insecurity (43–45). Second, CVD prevention through dietary practices should be a primary focus of a BP management intervention. Pharmacologic interventions can improve control of HTN and other CVD risk factors, but medications alone are sub-optimal and AI/AN adults with HTN are less likely than other racial groups to take anti-hypertensive medications (46). Further, medications do not address poor nutrition as a potential root cause of HTN. Benefits of the DASH diet have been demonstrated in primarily White and Black populations, but the DASH diet has never been tested with AI/AN participants. Given the unique historical and modern influences on the health of AI/AN communities, independent trials testing the effectiveness of interventions with AI/AN participants are warranted. Finally, an intervention needs to be relatively low cost to maximize accessibility and reach. One randomized trial of a clinic-based intervention in rural, AI/AN adults with diabetes showed that intensive BP management slowed or even reversed carotid intima-media thickening (47, 48). However, this approach is prohibitively expensive and logistically demanding for patients who lack adequate health insurance, reliable transportation, or ready access to care, all of which are common challenges for urban AI/AN adults.

The Native Opportunities to Stop Hypertension (NOSH) intervention is formulated after a recent DASH-based intervention, “Five Plus Nuts and Beans,” designed for urban Black adults, who experience many of the same barriers to healthy diet as urban AI/AN adults. In the Five Plus Nuts and Beans intervention, 120 participants were randomized to receive educational material on the DASH diet plus a weekly stipend for grocery delivery (control), or to receive DASH-oriented nutritional counseling and support from a registered dietitian (RD) plus a weekly stipend for heart-healthy grocery delivery (intervention). Participants in the intervention reported increased fruit and vegetable consumption and had improved urine potassium and sodium (49). Modeled after this intervention, the NOSH intervention includes a culturally-tailored DASH telenutrition curriculum that: emphasizes low sodium intake; emphasizes consumption of available traditional AI/AN foods; facilitates problem solving and provides strategies for maintaining healthy eating habits; and offers eight $30 weekly grocery orders. Traditional AI/AN foods, such as corn, salmon, trout, beans, and squash, are ubiquitous in contemporary grocery stores. However, recognition and acknowledgement that commonly stocked foods in grocery stores have their origins with Indigenous people is rare. By adapting various aspects of the Five Plus Nuts and Beans program and strong evidence-based dietary recommendations, the probability of effectiveness is increased.

Despite the growing number of urban AI/AN adults and longstanding disparities in CVD prevalence, no clinical trial has tested a therapeutic dietary intervention aimed to improve BP control among AI/AN with physician-diagnosed HTN. Thus, the NOSH study will evaluate an RD-delivered, adapted DASH intervention with urban AI/AN adults who receive care at one of three urban Indian clinics. In the NOSH intervention, weekly grocery orders will be complemented by nutrition counseling to promote locally-available food sources and motivate participants to adopt the DASH eating plan. This paper describes the NOSH randomized controlled trial to improve BP control among AI/AN adults. This study was approved by the Washington State University Institutional Review Board (#16118), the University of Oklahoma Institutional Review Board (#665427) and the Chickasaw Nation Institutional Review Board and has been registered with ClinicalTrials.gov (NCT02796313).

## 2. Materials and methods

### 2.1. Study aims

**Figure 1:**
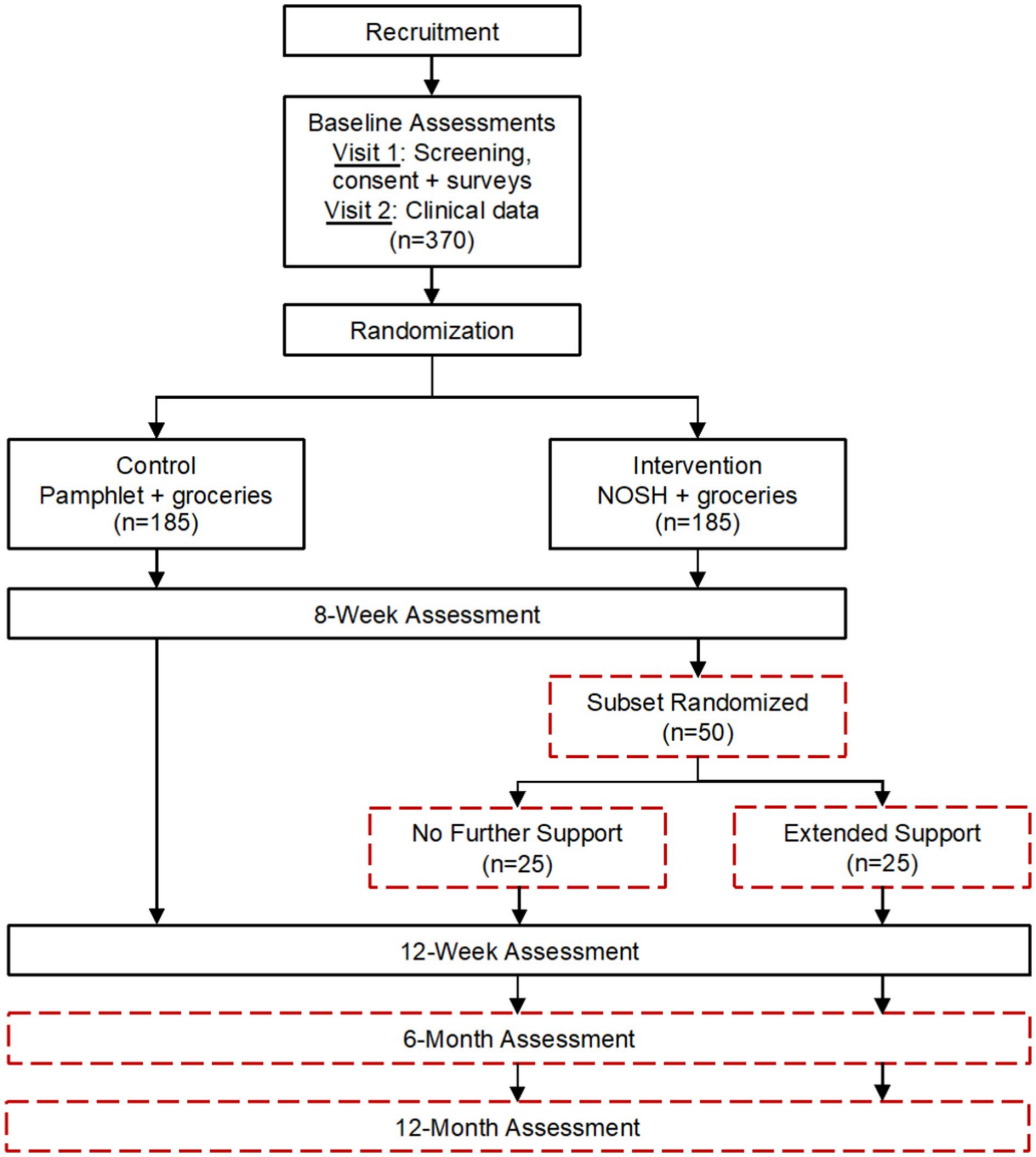
Native Opportunities to Stop Hypertension (NOSH) randomized controlled trial flow diagram and projected sample sizes.

The NOSH study aims are to: (1) evaluate the effect of the NOSH intervention on BP and secondary outcomes in urban AI/AN adults with HTN; and (2) conduct a pilot study after the intervention concludes to evaluate extended support from an RD for an additional 6 weeks among a subset of participants. NOSH is a randomized, wait-list control trial (Figure 1). After completion of the two baseline visits, participants will be randomized into either the intervention or waitlist control group. Waitlist control condition participants will receive a brochure about the benefits of a low-sodium diet and complete weekly phone calls to collect BP readings and place grocery orders. After completion of data collection (12-months post-baseline), participants will be offered the intervention. The intervention group will receive the intervention and groceries (described in 2.6). At week 8, if participants are interested in participating in the pilot study, they will be randomized into either the pilot study control or extended support (intervention) groups. It is hypothesized that NOSH will result in improved BP management and secondary outcomes compared to the control condition. For the extended support pilot study, it is hypothesized that BP will attenuate toward baseline in both groups of the pilot study, but extended support will lead to more sustained improvement. The study design used to address these aims is shown in Figure 1.

### 2.2. Setting

The trial will be implemented in two urban clinics in Washington state and one urban clinic in Oklahoma. Urban AI/AN communities vary in food-related cultural norms and values, as well as in local resources for obtaining healthy food; therefore, the inclusion of three sites will support generalizability of results and feasibility of dissemination across multiple diverse sites. One Washington clinic recently recorded annual visits from 400 unique HTN patients, while the other Washington clinic recently recorded 704, and the Oklahoma site recorded 1,897 patients; all sites serve at least 100 new patients with HTN annually. The NOSH study uses a participatory research approach to ensure the intervention is feasible within clinics and relevant to participants and that research findings will benefit the study populations (50). Across all sites, this study leverages the expertise of community-based nutrition and dietetics practitioners who each have direct practice experience within these AI/AN communities.

### 2.3. Participants

#### 2.3.1. Sample size

Data from previous DASH interventions (51, 52) was used to estimate a sample size of 185 participants per group required to detect at least a 5.3 mmHg difference in mean systolic BP at 8 weeks, and a 5.7 mmHg difference in the 12-week analysis excluding the 25 extended support pilot study participants. Power of 80%, alpha error rate of 0.05, and a standard deviation of 18 mmHg based on previous 8- or 9-week trials were assumed.

#### 2.3.2. Recruitment

A total of 370 participants will be recruited (approximately 120 from each site) with equal representation of men and women. At each clinic, separate lists of men and women in the target age range with physician-diagnosed HTN and at least two systolic readings ≥130 mmHg will be created. Each list will be randomly sequenced by the study biostatistician. Starting at the top of the lists, the research team will work with clinic staff to review medical charts for additional eligibility criteria. For patients who meet eligibility criteria, study staff will contact the primary care provider to secure approval for participation. Patients will be contacted by telephone and/or in-person at their next clinic visit to ascertain interest about the study and confirm eligibility. Eligible individuals will be scheduled for an initial baseline visit, ideally within 2 weeks of the in-person contact. Final determination of eligibility will occur at the second baseline visit based on an additional BP measure and compliance with study protocols (described below).

#### 2.3.3. Eligibility criteria

Participants must: (1) be at least 18 years old; (2) have had a clinical diagnosis of HTN for at least 1 year; (3) be on a stable regimen of antihypertensive medications for a minimum of 2 months OR not currently medicated, without anticipated changes for the duration of the study; (4) have average systolic BP ≥ 130 mmHg at the past two clinic visits and at the in-person screening visit; (5) have regular medical care and permission from their primary care provider to participate; (6) be physically and cognitively able to use the home BP monitoring device; and (7) be willing and able to follow all other study procedures. People will be ineligible if they: (1) experienced incident CVD or stroke within the previous 6 months; (2) have average diastolic BP ≥ 100 mmHg during the run-in period; (3) have a known diagnosis of secondary HTN (e.g., renal artery stenosis); (4) have diagnosed Stage 4 or 5 kidney disease; (5) have a recent history or high blood potassium due to taking certain medications that can raise potassium levels; (6) are participating in another health research study; (7) are currently or planning to become pregnant during the course of the study; or (8) are receiving treatment for cancer or another serious or terminal medical condition.

#### 2.3.4. Consent

The in-person screening will take place at the first baseline visit, which will occur within 2 weeks of initial screening for eligibility and interest. A research assistant will describe the study and risks of participation. The site study coordinator will also review the study and measure BP. For people whose average BP is within eligibility limits, the site study coordinator will administer the full informed consent protocol, explaining study goals, procedures, and potential risks, in addition to requesting Health Insurance Portability and Accountability Act (HIPAA) authorization to allow ongoing access to their medical records (HIPAA is meant to protect a patient from having their information disclosed without the individual’s consent or knowledge). Clinic staff will enter information pertinent to the patient’s care in the medical record notes as it is deemed appropriate and will document provider approval to participate in the study. These notes will be left to the discretion of clinic staff and will not be shared with the study team. Participants will provide written informed consent and HIPAA authorization.

For the extended support pilot study, at the 8-week appointment, while intervention group participants are completing their follow up data collection, the research assistant will access the next assignment on a randomized list created by the study biostatistician. If intervention group participants are interested in participating in the pilot study, they will undergo an additional informed consent process and be further randomized to one of the following two groups: (1) pilot study control, or (2) extended support (pilot study intervention). Potential participants will be clearly informed that their choice whether to participate in the pilot study will not affect their participation in the 12-week follow-up for the main trial.

### 2.4. Assessment procedures

Participants will complete four clinic visits (screening/baseline 1 visit, baseline 2 visit, week 8, and week 12) for the study. Two baseline visits are conducted to: (1) minimize the length of clinic visits by collecting surveys and clinical data on two separate dates, and (2) as a way of assessing compliance with study protocols. Participants are provided 24-h urine collection materials and the home BP monitor at the end of the first baseline visit. If they have completed the at-home urine and BP data collection when they return for the second baseline visit, they are considered compliant with study protocols. Compensation will be in the form of gift cards to local gas stations or grocery stores in the following amounts: second baseline visit ($50), 8- and 12-week visits ($50 each).

For the extended support pilot study, telephone interviews will be conducted to collect home BP data using the same study-provided devices at six and 9 months post-baseline. About 5 months post-baseline, pilot study participants will be contacted to schedule the 6-month data collection interview and provide re-training in use of the home BP cuff, if necessary. The 6- and 9-month interviews will be conducted by telephone by research staff who are masked to treatment group assignment. Staff will talk each participant through the process of accessing BP values stored in the cuff’s memory. Participants will read off values, and staff will enter them into a study database. Phone conversations will be recorded as electronic data files and erased after quality control checks have been completed.

### 2.5. Randomization

Treatment conditions will be randomly assigned after the participant completes the clinical data collection at the second baseline visit. The study staff will generate treatment assignments using the randomization function in the study’s REDCap database.

### 2.6. Intervention

The DASH diet is low in saturated fat, cholesterol, and total fat, focuses on fruits, vegetables, and fat-free or low-fat dairy products, and is rich in whole grains, fish, poultry, beans, seeds, and nuts. It minimizes intake of ultra-processed foods, including sweets, added sugars, and sugary beverages, as well as red meats compared to the typical American diet. Its unique composition of prescribed food groups results in a nutrition profile that is lower in sodium and rich in potassium, magnesium, and calcium. The intervention group will receive one in-person, tailored nutrition counseling visit at baseline and weekly 15-min telenutrition sessions in weeks 2–8 to provide additional DASH diet education and behavior change support.

After the final DASH session is complete, a questionnaire will be mailed to the participant in order to evaluate their satisfaction with the program. The 14-item anonymous questionnaire asks participants to rate their degree of agreement with statements such as, “The information about the DASH diet was easy to understand,” “It is difficult for me to do exactly what the nutrition staff recommended,” and “I will be able to follow the DASH diet and nutrition staff’s advice after the program.” Additional questions ask what participants liked about the program, what could have been improved or changed, what activities or information was most useful, and what diet and lifestyle changes were the most difficult to change.

During the extended support pilot study, the 25 intervention participants receiving extended support will continue to receive weekly 15-min phone consultations with the RD for an additional 6 weeks (weeks 9–14) after the end of the original 8-week study period. Consultations will provide support for continuing the DASH diet and will focus on overcoming barriers to obtaining and preparing healthy foods within the context of each person’s regular food budget and resources.

#### 2.6.1. In-person nutrition visit

Following randomization to the intervention group, participants will be scheduled for an initial appointment with an RD employed by the clinic and trained on all study procedures. This 55-min counseling visit will include: purpose of study visit (5 min.), energy needs calculation (5 min.), overview of the DASH diet based on calorie needs (15 min.), basic education on health risks of high BP and interpretation of current BP (4 min.), elicitation of patient perspective on which eating behaviors are affecting BP and ranking of readiness for behavior change (8 min.), co-creation of a customized eating plan (10 min.), and discussion of other lifestyle factors that may be affecting BP (e.g., tobacco use) (8 min.). Following the visit, the RD will assist participants with completing their first grocery order (10 min.). Participants will be provided with a daily eating goal handout for each DASH food group based on their estimated calorie needs. This handout was developed by a study co-investigator and RD (MSW), which was then reviewed and approved by RD community research staff. The handout includes references to hand images for each food group to estimate portion size (e.g., fist, palm, thumb) in addition to standard household measures (e.g., cup, Tablespoon). Participants will also receive a 56-page DASH diet booklet published by the NHLBI (53). During each weekly telephone session with the RD, specific pages of the booklet are referenced to facilitate participants’ gradual review of its content.

#### 2.6.2. Weekly telephone RD consultations

NOSH was developed to provide follow up medical nutrition therapy via telenutrition for the management of HTN. Typically delivered in person over multiple sessions, medical nutrition therapy is an evidence-based approach used by RDs to address chronic conditions, including HTN (54). However, multiple barriers, such as transportation and costs, preclude patients from accessing in-person healthcare, including medical nutrition therapy (55–57). The NOSH telenutrition curriculum will be delivered either over the phone or via password-protected secure Zoom platform, depending on participant preference, across eight weekly sessions by an RD or trained health educator under the direct supervision of an RD. Each session will begin with a check-in discussion about current BP monitoring at home with participant reporting of at-home readings (5 min.), interactive discussion about a particular eating goal within the DASH diet using a scripted, culturally-tailored telenutrition curriculum (10 min.), and placement of a $30 unrestricted grocery order (5 min.). Weekly topics will include: (1) seasoning without sodium; (2) fruits instead of processed sweets; (3) vegetables; (4) nuts, seeds, and beans; (5) whole grains; (6) healthy fats; (7) high-calcium dairy alternatives and low-fat dairy; and (8) lean meat, poultry, and fish. Telenutrition curriculum topics will relate to items that comprise the DASH diet score and include 2–3 learning objectives per session. Each session will incorporate motivational interviewing (58, 59) and provide time for the participant to reflect upon current eating behaviors, rate self-confidence in making needed eating behavior change, and develop a weekly action plan to improve dietary adherence within that session’s topic. This curriculum script for weekly telephone sessions was developed by a study co-investigator and RD (MSW), which was then reviewed, culturally tailored, and approved by RD community research staff. These staff members had various levels of experience that ranged from 7 years working with AI/AN adults to more than 20 years of experience working with AI/AN adults in their specific communities. At the end of each session, the RD will assist participants in placement of an unrestricted grocery order. A weekly list of suggested DASH foods specific to that week’s telenutrition topic was developed by the academic-community nutrition team for RD reference. Weekly food lists emphasized a variety of affordable perishable and non-perishable foods that could be accessed at the local grocery store.

#### 2.6.3. Registered dietitian training

RDs will be trained in patient-centered care and communications skills in three 2-h webinars. Sessions will be conducted by a master trainer who was involved in the DASH trials. Topics will include HTN, how to work with people with limited health literacy/numeracy skills, and how to address barriers to DASH and medication adherence. Webinars also will include a detailed review of the DASH diet and strategies to assess diet, set goals, provide advice, arrange follow-up, and monitor progress. Finally, a didactic session with role-playing will be completed to ensure training comprehension. Fidelity will be maintained through booster sessions conducted every 6 months.

### 2.7. Control condition

NOSH is a wait-list control trial. The control condition will receive a single printed brochure about the health benefits of a low-sodium diet after the second baseline visit, complete weekly phone calls to collect BP readings, and place $30 grocery orders. After all study data collection is complete, participants will be offered the same in-person, hour-long nutritional counseling session with the study RD that was provided to the intervention group, in addition to the eight weekly telenutrition calls without additional grocery credits provided.

### 2.8. Primary outcome

The primary study outcome will be systolic BP assessed at home as the average of three readings spaced 30 s apart using a Microlife 3BTO Plus (60). Participants will be instructed to take their BP at least two times a day (morning and evening) for 7 days before visits at baseline, 8 weeks post-baseline, and 12 weeks post-baseline. During the initial set up of the home BP monitors, measurements will be checked against clinic BP monitor measurements or manual measurements to ensure accuracy of home BP readings. For pilot study participants, home BP monitoring will also be conducted during the 6- and 9-months post-baseline interviews.

### 2.9. Secondary outcomes

#### 2.9.1. Blood pressure (clinic visits)

At the first baseline visit and at every subsequent clinic visit, systolic and diastolic BP will be measured on-site by auscultation using a mercury sphygmomanometer. A total of three BP measurements, separated by 30 s, will be obtained at each visit on the right arm of participants, using a cuff of appropriate size, after they rest quietly in the seated position for at least 5 min. If the participant indicates that there is a medical or post-surgical reason for not having the BP measured on the right arm (or if the right arm is missing), then BP will be measured with the cuff on the left arm.

#### 2.9.2. Body composition

Height and weight will be measured at the second baseline visit to calculate BMI as a continuous variable (kg/m^2^) and categorized to conventional thresholds (< 25 kg/m^2^ = normal; 25–29 kg/m^2^ = overweight; ≥ 30 kg/m^2^ = obese). Weight will be measured and at each following clinic visit to calculate BMI. Participants will be asked to remove shoes, heavier outerwear, and to empty heavy items from their pockets before weighing. If a participant is unable to remove shoes or heavier outerwear, two pounds will be removed from weight.

#### 2.9.3. Lipids

Fingerstick blood samples will be acquired at the second baseline visit and at each follow-up clinic visit to assess blood lipids. Blood lipids will be measured as total cholesterol, HDL cholesterol, LDL cholesterol, and triglycerides (all mg/dL), using the Cholestech portable analyzer (61). Hyperlipidemia will be defined as LDL cholesterol >130 mg/dL.

#### 2.9.4. Dietary intake

Two methods will be used to evaluate dietary intake at the second baseline visit and at each of the follow-up clinic visits. First, at the baseline, 8-, and 12-week assessments, the site study coordinator will assist participants with completing a food recall using the Automated Self-Administered 24-Hour (ASA24®) Dietary Assessment Tool developed by the National Cancer Institute (62). This website guides participants through a 24-h food recall and provides an animated guide and audio and visual cues that enhance use in low-literacy populations. Respondents report frequency of eating occasions and times of consumption. Detailed prompts assess food preparation, portion size, food source, and additions to food items. Dietary intake estimated through the ASA24 will be used the primary method for estimating changes in dietary intake for various components of the DASH diet across study timepoints and between intervention groups. Second, at baseline, self-reported consumption of fats, fruits, and vegetables will be assessed using the validated Block Food Frequency Questionnaire, which reflects dietary patterns during the past year (63). To reduce respondent burden at 8- and 12-week assessments, participants will complete the Fruit and Vegetable Screener, a validated, self-report measure of daily fruit and vegetable intake (64) and the Fat Screener, a questionnaire which assesses percent of energy intake from fat (65). Respondents are asked to indicate how often during the prior month they ate a variety of fruits and vegetables and a variety of food products that are high in fat, respectively. The (65) These food frequency questionnaires will be used to descriptively characterize any dietary changes indicated by the ASA24.

#### 2.9.5. Urinary sodium and potassium

A 24-h urine collection and a spot urine collection will be used to measure electrolytes at the second baseline visit and each follow-up clinic visit by ion-selective potentiometric methods and urine creatinine by a standardized enzymatic assay on the Vitros 5.1 platform (66). Collections with total volume < 450 mL/d or creatinine <10 mg/kg/d will be considered incomplete (67). Participants will be instructed in proper technique and provided with two 3 L wide-mouthed, labelled, screw-top plastic urine jugs along with a collection device (urinal or hat). Twenty-four-hour urine collections will start following first-morning void on the day prior to the study visit and end with inclusion of first morning void on the visit day, and the spot urine will be collected in-person at the visit. Urine volume will be measured using a graduated cylinder, with aliquots frozen at -80C prior to assay.

#### 2.9.6. Heart disease and stroke risk scores

For participants who have not experienced previous CHD or stroke events, the 10-year risk of incident CHD (68) or stroke will be estimated using prediction algorithms specific to AI/AN adults. Both risk scores were developed by the Strong Heart Study, a large prospective cohort study of CVD in AI/AN adults from three distinct geographic populations (including Oklahoma) (68). The scores are based on data collected during baseline and at each clinic visit (age, HTN medications, systolic BP, blood lipids, diabetes, smoking status, and albuminuria).

### 2.10. Covariate measures

#### 2.10.1. Medications

Participants will be instructed to bring all current medications (prescription, over the counter, and herbal or vitamin supplements) to each clinic visit. The site study coordinator will review and record each participant’s medications and will query the patient’s medical records. Current antihypertensive medications will be documented and coded using a protocol from an ongoing study of cerebrovascular disease among American Indians (69).

#### 2.10.2. Smoking and alcohol use

Tobacco and alcohol use at each clinic visit will be assessed with questions previously used in rural AI/AN populations (70). Smoking questions will distinguish current, past, and never smokers, including number of cigarettes smoked per day and age at initiation or cessation. Use of smokeless tobacco will not be assessed for this study. Alcohol consumption will be assessed with questions asking about frequency and type of current and past consumption of alcoholic beverages, after a standard definition and reference images for the equivalent of one alcoholic beverage is provided.

#### 2.10.3. Comorbid conditions

We will document prevalent diabetes, stroke, or CVD (congestive heart failure, myocardial infarction, or CHD) via patient self-report at the first baseline visit and medical chart abstraction. Urine micro- and macro-albuminuria will be categorized based on lab values where a participant’s albumin: creatine ratio is 30–299 mg/g or ≥ 300 mg/g, respectively (71).

#### 2.10.4. Demographic and administrative

Sex, age in years, completed education, current employment, and marital status will be collected at the first baseline visit. Documented administrative variables will include clinic site, study arm (control, intervention), and primary care provider (anonymous ID number, used only for statistical analysis).

### 2.11. Monitoring

The participants’ healthcare providers will be alerted that their patients are adopting an antihypertensive diet. In addition to monitoring provided by each participant’s primary healthcare provider, a Data Safety Monitoring Board (DSMB) will be organized to monitor the safety of all study participants. The DSMB will be responsible for safeguarding the interests of study participants, assessing the safety and efficacy of study procedures, ensuring data quality, and for monitoring the overall conduct of the study. The DSMB will be comprised of independent, doctorate-level professionals in the fields of medicine and AI/AN health. They will provide recommendations to the lead researcher, specifically related to starting, continuing, and stopping the study. In addition, the DSMB will be asked to provide recommendations, as appropriate, about: efficacy of the study intervention; benefit/risk ratio of procedures and participant burden; selection, recruitment, and retention of participants; adherence to protocol requirements; completeness, quality, and analysis of measurements; data and statistical analysis plan; amendments to the study protocol and consent forms; performance of individual study sites and core lab; adverse and serious adverse events; and participant safety. Meetings will be held once per year, with additional meetings or conference calls scheduled as needed. The lead researcher will promptly report all protocol deviations or unexpected, serious adverse events to the Washington State University Institutional Review Board and other relevant review boards, as per their protocols.

### 2.12. Data analysis

All data collected on-site will be scanned and uploaded to a secure server and entered into a REDCap database (72). Data will be reviewed by the biostatistician monthly to quickly identify data quality issues. For quality control, data from 10% of randomly selected participants will be entered twice. An intention-to-treat analysis will be used. Success of randomization will be assessed by using t-tests and chi-squared tests to compare baseline variables in the intervention and control groups. All inferential results will be presented as point estimates with 95% confidence intervals. Data analysis will be performed using Stata 14 (StataCorp LP, College Station TX, 2013).

Linear regression will be used to compare primary and secondary outcomes between the intervention and control groups at 8- or 12-week post-baseline assessments, with treatment group, baseline value, and study site as the independent variables. Since there may be differences between the study sites regarding availability of healthy food at local stores, an interaction term between intervention and site will be included. If the interaction is significant, all analyzes will be repeated after stratifying by site. Sensitivity analyzes will adjust for variables that appear unbalanced between groups at baseline. All 370 participants will be included in the analysis of data from the 8-week visit, but the 25 pilot study extended support (intervention group) participants will be excluded from analyzes involving data from the 12-week visit. Next, generalized estimating equations will be used to simultaneously assess outcome values measured at 8 and 12 weeks. To assess effectiveness of the extended support pilot study, a generalized estimating equation will be used to analyze data for all four time points (8 weeks, 12 weeks, 6 months, and 12 months), with independent indicators for each time point that do not impose a linear trend on the outcome. These models adjust standard error estimates to properly account for within-person correlation in the outcome values over time.

## 3. Discussion

Developing an effective diet-based intervention focused on decreasing CVD risk among urban AI/AN adults is of critical public health importance. DASH-based telenutrition interventions can be enacted within or external to a healthcare system, reducing barriers to access preventive care. Adding aspects to the program, like home food delivery, might make accessing fresh produce easier for urban AI/ANs as well as promote nutritional security (73). Creating evidence-based, culturally-adapted interventions that are relevant and engaging for AI/AN individuals could be instrumental to address the disproportionate burden of CVD morbidity and mortality experienced by AI/AN populations.

This study protocol has several notable strengths. First, to the authors knowledge, no previous dietary intervention specifically developed for HTN management has ever been conducted in AI/AN adults, urban or rural. If effective, this study can be scaled up to address the disproportionate burden of CVD among AI/AN adults (5, 74, 75), in which HTN has been widely implicated (76). Second, because sites can tailor the intervention to their unique communities, while maintaining core DASH components, this is an effectiveness trial. By contrast, clinical trials of non-pharmacological therapies, while optimizing the effect of an intervention, are conducted under ideal conditions of monitoring and compliance, which are typically not desirable or feasible in diverse AI/AN community settings. Third, although a focus on traditional foods is not a typical component of DASH, the addition of this cultural tailoring was appealing and important to study partners. Lost access to traditional foods and food systems is believed to contribute to lower dietary diversity and greater CVD disparities among AI/AN communities (24). Fourth, the use of home BP monitoring is significant. Home monitoring not only provides better diagnosis and risk prediction in HTN, but may also improve treatment by engaging patients (77), thereby improving dietary adherence and study retention. Fifth, DASH trials have far-reaching implications for clinical practice and public health. Adherence to the DASH diet lowers BP to the same extent as the five antihypertensive medications used as monotherapy in the Veterans Affairs Cooperative Study (78). For patients with HTN, the DASH diet is highly recommended as a potent adjunct to pharmacologic treatment and strengthens the likelihood of the intervention’s effectiveness. Sixth, this study contributes to the growing field of telenutrition, which is considered an important strategy for reducing access barriers to medical nutrition therapy. Lastly, the provision of grocery delivery may reduce barriers to access food items, encourage participants to try new foods, and direct coaching when making food purchasing decisions.

This study is not without limitations. Diet is subject to contextual effects beyond the individual and clinic, with family- and community-level influences on food choices and food access that must also be addressed (79). Therefore, lifestyle interventions in tight-knit AI/AN communities risk contamination if people in the intervention group influence the dietary choices of people in the control group. However, the study team does not expect substantial contamination, given that the study will be conducted in large urban areas, where study participants are not likely to interact directly with other participants. This limitation can be avoided only in a community-randomized trial, which is scientifically premature and beyond the scope of the current research.

If the trial is effective, NOSH will be an immediately scalable, inexpensive, and safe approach to treat HTN in urban AI/AN communities. Because most AI/AN adults live in urban settings, the widespread implementation of an effective program has the potential to dramatically improve public health of millions of AI/AN households. Further, since participants are not required to use medication for BP control, the target population includes people who have been unsuccessful at lifestyle change or are otherwise untreated for Stage I HTN. Given widespread barriers to healthcare for urban AI/AN adults, this innovation magnifies the potential population-level impact. If the program is effective, the two partner sites will start integrating the DASH diet into usual care. The more burdensome measurements taken for this effectiveness trial can be simplified to align with current clinical practice and may eventually be implemented by innovative mobile health technologies. Ongoing research probes the ability of smartphone apps to help patients adhere to the DASH diet. The NOSH intervention is inexpensive and easily tailored to community resources, such as community cooking events and neighborhood gardens that emphasize traditional foods. Because the DASH diet has been successfully used in clinical studies with other minority groups, we believe it can be readily disseminated to Native healthcare facilities across the United States.

## Data availability statement

The original contributions presented in the study are included in the article/supplementary material, further inquiries can be directed to the corresponding author.

## Ethics statement

This study was approved by the Washington State University Institutional Review Board (#16118), the University of Oklahoma Institutional Review Board (#665427) and the Chickasaw Nation Institutional Review Board and has been registered with ClinicalTrials.gov (NCT02796313).

## Author contributions

KS, VJ, and DB conceptualized the study design. MW designed the NOSH curriculum and co-led training of research staff on dietary assessment. KS, CN, MW, KN, TT, and AJ drafted the manuscript. CN supported data visualization. All authors reviewed and approved the final manuscript.

## Funding

This research is supported by funding from the National Heart, Lung, and Blood Institute at the National Institutes of Health, under award number R01HL126578. The funding body had no role in study design, data collection, analysis, or interpretation.

## Conflict of interest

The authors declare that the research was conducted in the absence of any commercial or financial relationships that could be construed as a potential conflict of interest.

## Publisher’s note

All claims expressed in this article are solely those of the authors and do not necessarily represent those of their affiliated organizations, or those of the publisher, the editors and the reviewers. Any product that may be evaluated in this article, or claim that may be made by its manufacturer, is not guaranteed or endorsed by the publisher.
